# Physical Activity, Mental Health, and Well-Being in Very Pre-Term and Term Born Adolescents: An Individual Participant Data Meta-Analysis of Two Accelerometry Studies

**DOI:** 10.3390/ijerph18041735

**Published:** 2021-02-10

**Authors:** Asteria Brylka, Dieter Wolke, Sebastian Ludyga, Ayten Bilgin, Juliane Spiegler, Hayley Trower, Anna Gkiouleka, Markus Gerber, Serge Brand, Alexander Grob, Peter Weber, Kati Heinonen, Eero Kajantie, Katri Räikkönen, Sakari Lemola

**Affiliations:** 1 Department of Psychology, University of Warwick, Coventry CV4 7AL, UK; asteria.brylka@gmail.com (A.B.); D.Wolke@warwick.ac.uk (D.W.); A.Bilgin@kent.ac.uk (A.B.); uni@dr-spiegler.de (J.S.); Hayley.Trower@warwick.ac.uk (H.T.); annagiouleka@gmail.com (A.G.); 2 Department of Sport, Exercise and Health, Sport Sciences Section, University of Basel, CH-4052 Basel, Switzerland; sebastian.ludyga@unibas.ch (S.L.); markus.gerber@unibas.ch (M.G.); Serge.Brand@upk.ch (S.B.); 3 School of Psychology, University of Kent, Canterbury CT2 7NP, UK; 4 Department of Paediatrics, University of Lübeck, 23562 Lübeck, Germany; 5 Psychiatric Clinics, Center for Affective, Stress, and Sleep Disorders, University of Basel, CH-4002 Basel, Switzerland; 6 Sleep Disorders Research Center, Kermanshah University of Medical Sciences (KUMS), Kermanshah 6715847141, Iran; 7 Substance Abuse Prevention Research Center, Health Institute, Kermanshah University of Medical Sciences (KUMS), Kermanshah 6715847141, Iran; 8 School of Medicine, Tehran University of Medical Sciences (TUMS), Tehran 1417863181, Iran; 9 Department of Psychology, University of Basel, CH-4055 Basel, Switzerland; alexander.grob@unibas.ch; 10 Division of Neuropediatrics and Developmental Medicine, University Children’s Hospital Basel, CH-4056 Basel, Switzerland; peter.weber@unibas.ch; 11 Department of Psychology & Logopedics, University of Helsinki, FI-00014 Helsinki, Finland; kati.heinonen-tuomaala@tuni.fi (K.H.); katri.raikkonen@helsinki.fi (K.R.); 12 Psychology/Welfare Sciences, Faculty of Social Sciences, Tampere University, FI-33720 Tampere, Finland; 13 National Institute of Health and Welfare, FI-00271 Helsinki, Finland; eero.kajantie@thl.fi; 14 PEDEGO Research Unit, Medical Research Center Oulu, Oulu University Hospital and University of Oulu, FI-90014 Oulu, Finland; 15 Children’s Hospital, Helsinki University Hospital and the University of Helsinki, FI-00014 Helsinki, Finland; 16 Department of Psychology, Bielefeld University, 33501 Bielefeld, Germany

**Keywords:** physical activity, mental health, well-being, preterm birth, adolescence, accelerometry

## Abstract

This study examined whether physical activity is associated with better mental health and well-being among very preterm (≤32 weeks) and term born (≥37 weeks) adolescents alike or whether the associations are stronger in either of the groups. Physical activity was measured with accelerometry in children born very preterm and at term in two cohorts, the Basel Study of Preterm Children (BSPC; 40 adolescents born ≤32 weeks of gestation and 59 term born controls aged 12.3 years) and the Millennium Cohort Study (MCS; 45 adolescents born ≤32 weeks of gestation and 3137 term born controls aged 14.2 years on average). In both cohorts, emotional and behavioral problems were mother-reported using the Strengths and Difficulties Questionnaire. Subjective well-being was self-reported using the Kidscreen-52 Questionnaire in the BSPC and single items in the MCS. Hierarchical regressions with ‘preterm status × physical activity’-interaction effects were subjected to individual participant data (IPD) meta-analysis. IPD meta-analysis showed that higher levels of physical activity were associated with lower levels of peer problems, and higher levels of psychological well-being, better self-perception/body image, and school related well-being. Overall, the effect-sizes were small and the associations did not differ significantly between very preterm and term born adolescents. Future research may examine the mechanisms behind effects of physical activity on mental health and wellbeing in adolescence as well as which type of physical activity might be most beneficial for term and preterm born children.

## 1. Introduction

Children and adults born very preterm or with very low birth weight have an increased risk of neurodevelopmental and mental health problems [1,2]. During adolescence and young adulthood, those born very preterm are often described as being more socially withdrawn and having problems with peers [3]. While prematurity increases the risk of adverse outcomes, many very preterm born children and adolescents do not have any symptoms of mental health problems, showing resilience in the face of complications and adversity [4]. However, research on resilience factors among very preterm children and adolescents is rather scarce. Existing research has identified sensitive parenting in childhood [5] as a potential resilience factor for academic achievement among very preterm children (i.e., in very preterm children sensitive parenting was more strongly associated with academic outcomes than in term born children). By contrast, a recent study reported that very preterm children may be more strongly affected by risk factors for mental health such as family dysfunction, parental mental health problems, and peer victimisation while not benefitting as much from potential resilience factors such as number of close friends or sensitive parental care compared to term born children [6].

Physical activity has been suggested to promote mental health and well-being among children and adolescents in the general population [7,8,9]. Furthermore, it has been linked to increased happiness [10], positive affect [11], improved facial emotion recognition [12], and decreased levels of anxiety, depressive symptoms, and socio-emotional difficulties [10,13]. In particular, it has been suggested that physical activity plays an important role for adolescents in building a favourable self-image and self-esteem, and in decreasing social inhibition and anxiety by evoking positive social feedback and recognition from peers [13,14].

Very preterm children tend to have more physical and motor limitations [15,16] and show reduced pulmonary function, aerobic capacity, and muscle strength [17]. Despite this, no difference in physical activity levels has been reported during adolescence between very preterm and term born children [18,19,20], although lower levels of physical activity have been observed in young adults [21,22]. However, research is scarce on whether physical activity has similar benefits for mental health and well-being among very preterm children and adolescents as it has among their term born peers [23]. As very preterm children are often reported to show higher levels of emotional and peer problems, increased physical activity may particularly benefit their self-image and recognition from peers and decrease social inhibition.

**Figure 1:**
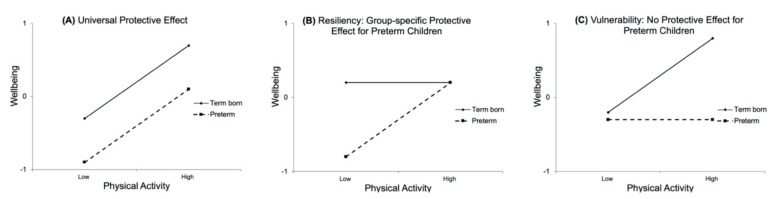
Three competing hypothetical models of the impact of physical activity in very preterm and term born adolescents. (**A**) Universal Protective Effect; (**B**) Resiliency: Group-specific Protective Effect for Preterm Children; (**C**) Vulnerability: No protective Effect for Preterm Children.

The objective of the current study was to examine three competing explanatory models regarding how physical activity may impact on very preterm born adolescents’ mental health and well-being (Figure 1). The first model (“(A) Universal Protective Effect”) proposes that physical activity protects very preterm and term born adolescents alike from lower mental health and well-being; the second model (“(B) Resiliency: Group-specific Protective Effect for Preterm Children”) proposes that physical activity is a resiliency factor that particularly benefits very preterm adolescents; while the third model (“Vulnerability: No protective Effect for Preterm Children”) proposes that very preterm adolescents benefit less from physical activity than those born at term [6]. While the first model (Model A) involves a main effect (i.e., physical activity showing the same association in both groups), the other two models describe moderation effects involving a stronger (Model B) or weaker (Model C) positive association among very preterm adolescents in comparison to term born adolescents. These models are tested using physical activity objectively assessed with accelerometry, mother-reported behavioral and emotional problems, and child-reported well-being in two samples of very preterm born adolescents and term born controls. Physical activity was assessed with accelerometry to avoid the common-method bias, which may inflate associations when both predictor and outcome are reported by the same person [24].

## 2. Methods

### 2.1. Participants

The Basel Study of Preterm Children (BSPC) is a cohort study of very preterm children and term born controls. The very preterm sub-sample was recruited from an initial cohort of 260 prematurely born children, who were treated at the University Children’s Hospital of Basel, Switzerland, between June 2001 and December 2006, and for whom gestational age was retrieved from hospital records [25]. Cross-sectional data used for the current study come from the third wave of the BSPC collected between February 2016 and October 2017, when the children were 11 to 15-year old (average age = 12.2 years, *SD* = 1.1). In total, 40 children and adolescents born very preterm (≤32 weeks and 0 days of gestation) had sufficient days of valid accelerometry recordings to be included (i.e., at least 4 days with a wear time of at least 70% including one weekend day). The control sub-sample included 59 term born participants, who were originally recruited through official birth notifications (≥37 weeks and 0 days of gestation; also including post-terms) as an age and sex matched comparison group in the first wave of the BSPC when children were 8 years old on average [25].

The Millennium Cohort Study (MCS) is a representative longitudinal study of 18,818 infants born in the UK [26]. A random sample was drawn from Child Benefit registers of infants born in the UK, between September 2000 and January 2002, and who were living in the UK at the age of 9 months. Parents were interviewed for the first time when the children were aged 9 months (survey 1), and again at 14 years (survey 6). Validity of parent-reported gestational age at 9 months after birth has been reported by Poulsen and colleagues [27]. Cross-sectional data at wave 6 were used for the current study, when participants were 14.2 years old (*SD* = 0.3). Data of twins and triplets were included in the analysis. Detailed analysis of sociodemographic data and a flow chart of study participation of different gestational age groups have been published before [20]. In total, 10,337 MCS members had been invited for the accelerometry protocol. After excluding 6258 participants with insufficient accelerometer data (i.e., participants with a wear time of 70% or less on one of two accelerometry assessment days), and 292 with missing data on gestational age or gestational age ranging from 32 + 1 to 36 + 6 weeks, the study sample included 45 very preterm born adolescents (≤32 weeks and 0 days of gestation) and 3137 term born participants (≥37 weeks and 0 days of gestation; also including post-terms). Very preterm children were not more likely to drop out of the analysis compared to their term born counterparts (participation rate among very preterm adolescents was 17.8% compared to 18.7% among those born at term; (*x*^2^(1) = 0.13, *p* = 0.72)).

Sample characteristics of very preterm and term born adolescents are shown in Table 1 for both cohorts.

### 2.2. Ethical Considerations

Ethical approval from the respective ethic committees (BSPC: Ethikkommission Nordwest-und Zentralschweiz, reference number 122/11; MCS: London-Hampstead Research Ethics Committee, REC reference 14/LO/0868) were obtained and participants gave written informed consent.

### 2.3. Measures

#### 2.3.1. Physical Activity

In the BSPC, physical activity was measured over seven consecutive days with triaxial accelerometers (ActiGraph LLC; Pensacola, FL, USA) worn on the wrist of the non-dominant hand throughout the day. ActiLife software 6.13.3 (ActiGraph LLC) was used for the initialization of accelerometers, download, and processing of collected data. Prior to analyses, wear time validation was performed with the Troiano and colleagues′ [28] algorithm implemented in the program that defines non-wear time by an interval of 60 or more consecutive minutes of zero activity intensity counts, with allowance for one to two minutes of counts between 0 and 100. Recorded days with a non-wear time of 30% or higher were excluded from the analyses. A dataset was considered valid, if data covered at least four days after controlling for non-wear time, and if one of these days was a valid weekend day [29]. Physical activity on valid days was coded into number of minutes of sedentary, light, moderate, and vigorous physical activity using counts per minute cut-points, which have been validated in children [30]. Cut-off points for moderate and vigorous physical activity were 3600 counts/min and 6200 counts/min, respectively. The level of objective physical activity per week was calculated by summing minutes of vigorous and moderate physical activity performed by the participants divided by the number of days of accelerometry data collection and multiplied by 7. To use physical activity in cross-cohort analysis, the variable was z-standardized.

In the MCS, objective physical activity was measured using GENEActiv Original accelerometer devices worn on the wrist of the non-dominant hand on one day during the week and one weekend day calculating Euclidean Norm Minus One (ENMO). Total number of minutes are indicated summing up 1-min epochs with ENMO > 100 mg [31]. This variable representing the total number of minutes of moderate to vigorous physical activity was highly correlated with other definitions of moderate-vigorous physical activity (e.g., it correlated at r = 0.85 (*p* < 0.001) with moderate to vigorous physical activity defined as bouts of 5-min windows with 80% of time spent in moderate-vigorous physical activity). To calculate a weekly average, weekday physical activity was multiplied by 5 and weekend physical activity was multiplied by 2 before summing the two values. To use physical activity in cross-cohort analysis, the variable was z-standardized.

#### 2.3.2. Behavioral and Emotional Problems

Mothers reported on behavioral and emotional problems in both the BSPC and the MCS using 20 items of the Strengths & Difficulties Questionnaire (SDQ) [32]. The response scale for each item ranged from zero (*not true*) to two (*certainly true*). The overall score and the sub-scores for the four domains of emotional symptoms (five items), conduct problems (five items), hyperactivity and inattention (five items), and peer problems (five items) were used in analyses. Higher scores correspond to more severe behavioral and emotional problems. Reliability coefficients (α) are shown in Table 1.

#### 2.3.3. Positive Well-Being

Positive well-being was self-reported with the Kidscreen-52 [33] in the Basel Study of Preterm Children (BSPC), and with single items in the Millennium Cohort Study (MCS). Facets of positive well-being measured in both studies included *psychological well-being* (Kidscreen-52-example item: “Have you been satisfied with your life?”, MCS-single item: “How do you feel about your life as a whole?”), self-perception (Kidscreen-52-example item: “Did you worry about your appearance?”, MCS-single item: “How do you feel about the way you look?”), peer relations (Kidscreen-52-example item: “Could you rely on your friends?”, MCS-single item: “How do you feel about your friends?”), school related well-being (Kidscreen-52-example item: “Have you been happy at school?”, MCS-single item: “How do you feel about the school you go to?”). The overall well-being scale involved all Kidscreen52 items in the BSPC and a mean score of the MCS-single item scales. The Kidscreen-52 included five-point Likert scales, while the MCS-single items included a seven-point Likert scale (1 = ‘completely happy’ to 7 = ‘not at all happy’); all scales were coded such that higher values denoted higher levels of well-being.

#### 2.3.4. Covariates 

The following covariates were included in the analysis: participants’ age at the assessment of the outcomes, sex, neurosensory impairment, ethnic minority group membership, and parental education. Neurosensory impairment was defined as parent-reported severe visual impairments, hearing deficits, or motor impairments (including cerebral palsy), or cognitive ability scores of at least three SDs below the population average. Cognitive ability scores were assessed with the Wechsler Intelligence Scale for Children-IV in BSPC, at an average age of 10 years (during the second study wave of the BSPC), and with the British Ability Scales (Picture Similarity and Pattern Construction) in the MCS, at the age of 5 years. Ethnic minority group membership was measured with adolescents’ first language, which was used as a proxy (0 = German; 1 = other) in the BSPC, and with parent-reported ethnic minority status (0 = white British; 1 = other) in the MCS. Parental education was defined by the highest educational level of either parent (0 = no parent had tertiary education; 1 = either the mother or the father had tertiary education).

### 2.4. Statistical Analysis

Preliminary analyses were conducted separately for both cohorts and involved comparing physical activity, behavioral and emotional problems, and well-being between very preterm and term born adolescents as well as examining Pearson’s correlations between physical activity and covariates. This was followed by random-effects meta-analysis to compare pooled means of physical activity, behavioral and emotional problems, and well-being of very preterm and term born adolescents across the two cohorts.

As the main analysis, two-step individual participant data (IPD) meta-analysis was conducted. In the first stage, following the procedure of Aiken and West [34], hierarchical regression analyses were conducted with physical activity, preterm status, and their interaction term (physical activity × preterm status) as predictors added to the regression model in consecutive steps. All analyses were controlled for sex, age at assessment, neurosensory impairment, ethnic minority group membership, and parental education. Outcome variables were mother-reported behavioral and emotional problems or child-reported well-being. Outcome variables and physical activity were z-standardized prior to analysis to allow for comparisons between the two cohorts and unstandardized regression coefficients are displayed. These analyses were conducted using SPSS (version 24; IBM Corporation, Armonk, NY, USA).

In the second stage, two separate sets of meta-analyses were conducted for main effects of physical activity (i.e., regression coefficients for physical activity before interaction terms were added to the hierarchical regression models) and interaction effects (physical activity × preterm status). Pooled effects and 95% confidence intervals (CIs) were computed using the random-effects method with DerSimonian and Laird technique [35]. In all meta-analyses, between-study heterogeneity was tested using the Cochran’s *Q* statistic and quantified by the *I*^2^ value. Low heterogeneity was defined as an *I*^2^ value of 0–25%, moderate heterogeneity as an *I*^2^ of 25–75%, and high heterogeneity as an *I*^2^ of 75–100%. Two-stage random-effects IPD meta-analysis was conducted using the ‘ipdmetan’ command [36] in STATA (version 15.0; StataCorp, College Station, TX, USA). We applied a Bonferroni correction to account for multiple comparisons (*p* = 0.05/10 = 0.005).

We further conducted two sensitivity analyses. In the first sensitivity analysis, we repeated all analyses excluding those who were twins/triplets (as including these participants this could have resulted in skewed findings). In the second sensitivity analysis, we used propensity score matching using the ‘psmatch2′ command in STATA in the MCS cohort, where there was a large difference in the number of participants between the very preterm and term born groups, and repeated the regression analysis. Using propensity score matching has advantages including that it provides a more precise treatment effect by creating a balanced data set [37].

## 3. Results

### 3.1. Descriptive Statistics

Participants′ characteristics are presented in Table 1. In the BSPC sample, very preterm adolescents were less likely to have parents with tertiary education than term born adolescents. Moreover, very preterm adolescents were more likely to belong to ethnic minority groups in the MCS sample. Pooled mean differences based on random effects meta-analysis revealed significantly higher levels of emotional problems in very preterm compared to term born children (Std. mean difference = 0.26, *p* = 0.03; Supplementary Table S1). However, no further significant differences in mean levels of behavioral and emotional problems, well-being, and physical activity between the groups were found.

In the BSPC sample, physical activity correlated significantly with age (r = −0.42, *p* < 0.001) but not with sex, neurosensory impairment, ethnic minority group membership, and parental education (all *p*-values > 0.20). In the MCS sample, physical activity was associated with sex (r = −0.12, *p* < 0.001), ethnic group (r = −0.06, *p* = 0.002), and parental education (r = −0.06, *p* < 0.001), indicating lower physical activity among girls, participants with ethnic minority background, and with parents with lower educational levels, but was not correlated with age.

### 3.2. Individual Participant Data (IPD) Meta-Analysis

Table 2 displays the findings of the IPD meta-analysis. In the IPD meta-analysis of the main effects, physical activity was associated with lower levels of emotional symptoms (β = −0.048; 95% CI = −0.081–−0.014) and peer problems (β = −0.086; 95% CI = −0.120 –−0.053) but higher levels of hyperactivity/inattention (β = 0.066; 95% CI = 0.016–0.116). Physical activity was also associated with higher levels of overall well-being (β = 0.057; 95% CI = 0.022–0.091), psychological well-being (β = 0.053; 95% CI = 0.019–0.087), more favorable self-perception (β = 0.061; 95% CI = 0.028–0.094), and better school related well-being (β = 0.056; 95% CI = 0.022–0.090). Using a Bonferroni correction (*p* = 0.05/10 = 0.005), the associations between physical activity and hyperactivity/inattention and emotional problems were non-significant. No statistically significant heterogeneity existed between the study cohorts in the analyses of the main effects (*p*-values > 0.14). Findings remained the same in the sensitivity analysis repeating the analysis without the twin/triplet participants (i.e., all formerly significant associations remained significant at the Bonferroni corrected level of *p* < 0.005; Supplementary Table S2).

In the IPD meta-analysis of interaction effects the only significant association was between the physical activity × preterm status interaction and hyperactivity/inattention (β = −0.281; 95% CI = −0.557–−0.005), which became nonsignificant after correction for multiple comparisons. Apart from this association, there were no significant physical activity × preterm status interactions with any of the outcome variables. There was significant heterogeneity between the two samples in general and psychological well-being, which were not explained by female sex, minority ethnicity, and university level parental education (*Ps* > 0.05). For both domains, the heterogeneity was indicative of increased well-being among very preterm adolescents with higher physical activity in the BSPC sample, but of an inverse relationship for the MCS.

The findings of the hierarchical regression analyses for the two cohorts (i.e., the first stage of the IPD meta-analysis) are displayed in Table 3. Findings regarding the physical activity × preterm status interactions remained similar after using propensity score matching for the MCS cohort (data not shown).

## 4. Discussion

IPD meta-analysis for the two cohorts revealed that higher levels of physical activity were associated with lower levels of peer problems reported by mothers. Similarly, higher levels of physical activity were associated with higher overall well-being, psychological and school-related well-being and better self-perception and body image as reported by adolescents themselves. The association of physical activity with behavioral and emotional problems and well-being was not found to be different between very preterm born and term born adolescents. Thus, consistent with the first of the three competing explanatory models (“(A) Universal Protective Effect”), physical activity is similarly associated with behavioral and emotional problems and well-being among all adolescents. Very preterm adolescents were neither more vulnerable nor more susceptible to the effects of physical activity. However, the observed effect sizes were small.

It has been suggested that physical activity may have a positive effect on mental health and well-being among adolescents, as it may evoke positive social feedback and recognition from peers, which in turn improves self-image and decreases social inhibition and anxiety [13]. Very preterm adolescents and adults are more often socially withdrawn and anxious [3,38] as well as socially excluded and bullied by peers [2]. Activities that decrease social anxiety and enhance peer recognition, such as physical activity, were expected to particularly improve mental health and well-being in very preterm adolescents. However, the findings indicate that physical activity was associated with improved self-perception and decreased peer problems among both very preterm and term born adolescents.

Finally, the findings are consistent with studies indicating that during adolescence, those born very preterm show similar levels of physical activity as their term born counterparts [18,19,20], while differences may develop later in young adulthood when those born preterm at very low birth weight (<1500 g) report over 50% less physical activity than term born peers, measured by questionnaire, while no differences were seen when measured by accelerometry [21,22].

The following limitations of the study need to be mentioned. First, preliminary analyses only showed significant mean differences between very preterm and term born adolescents regarding emotional problems, while no further significant differences in behavioral and emotional problems and well-being were found in both cohorts. However, there is a large body of evidence suggesting that differences regarding attention difficulties and peer problems exist as well [1,38,39]. It is possible that the main analyses were affected as the cohorts included very preterm children with fewer attention difficulties and peer problems than expected. Relatedly, the two samples differ in how they were recruited and how representative they are for the entire population of very preterm and term born children. While it is conceivable that generally analyses based on the Millennium Cohort Study may lead to more generalizable conclusions, this may not necessarily transfer to analyses related to the relatively small subsample of very preterm children. Second, while IPD meta-analysis of two cohorts was used to increase statistical power, the overall number of very preterm adolescents studied was still relatively small and power to detect interaction effects was limited (observed power for the strongest interaction effect, i.e., regarding hyperactivity and inattention, was 0.72 before the Bonferroni correction). This is in contrast to the much larger number of term born adolescents particularly in the MCS, which allowed examining the main effects of physical activity with ample statistical power. Third, the two cohort studies used different accelerometry devices, different definitions for valid accelerometry recordings, and different scales to measure wellbeing. Relatedly, the accelerometers in both studies were worn on the wrist, while studies on physical activity often use waist-worn devices (e.g., [28,29]). Furthermore, while MCS recruited all participants early in life, the BSPC followed a case-control approach matching very preterm children with control children according to age and sex at the first study wave when the children were 8 years old. These differences may have increased heterogeneity of the findings between the two studies. Fourth, only a relatively small percentage of the original birth cohorts had valid accelerometry readings in adolescence. While in MCS the drop out was not selective for very preterm birth, a dropout analysis from birth to follow-up was not feasible for the BSPC because the comparison sample was recruited at the first follow-up time point at 8 years of age on average. Fifth, while the use of objective measures of physical activity may be regarded as a strength of the study, it may also involve a limitation. Accelerometry is better able to measure the number of steps, while it is not sensitive to other types of physical activity such as cycling or static exercise (see [40] for a discussion). It is possible that some types of exercise have more positive effect on well-being among adolescents than others, as it has been suggested for learning to play golf vs. playing soccer [41]. Particularly highly competitive team sports might also impose a threat to self-esteem and well-being for some adolescents. The current study does not inform us regarding the actual type of physical activity that has the greatest potential to positively affect mental health and well-being among very preterm and term born adolescents. Finally, causality cannot be inferred due to the observational cross-sectional design.

## 5. Conclusions

In conclusion, this study suggests that physical activity is associated with improved mental health and well-being for all adolescents, whether born full term or very preterm, although the effect size is small. It remains a task for future studies to determine whether distinct types and aspects of physical activity have a greater influence among very preterm or term born adolescents.

## Figures and Tables

**Table 1:** Sample characteristics of the very preterm and term born adolescents and reliability indices for study scales

	Basel Study of Preterm Children				Millennium Cohort Study				
	Very Preterm n = 40 N(%)/M(SD)	Control n = 59 N(%)/M(SD)	Very Preterm vs. Control	Cronbach’s α	Very Preterm n = 45 N(%)/M(SD)	Control n = 3137 N(%)/M(SD)	Very Preterm vs. Control	Cronbach’s α	Std. Pooled Mean Difference ^1^
Gestational age (weeks)	30.2 (2.1)	39.5 (1.5)	*p* < 0.001	-	29.5 (2.4)	39.6 (1.3)	*p* < 0.001	-	
Age at assessment (years)	12.3 (1.0)	12.1 (1.1)	*p* = 0.233	-	14.3 (0.4)	14.2 (0.3)	*p* = 0.130	-	
Sex (*n* Male)	21 (51.2)	31 (50.8)	*p* = 0.968	-	22 (48.9)	1438 (45.8)	*p* = 0.684	-	
Neurosensory impairment (*n*)	2 (4.9)	1 (1.6)	*p* = 0.563	-	2 (4.4)	56 (1.8)	*p* = 0.197	-	
Ethnic minority group (*n*)	10 (24.4)	7 (11.5)	*p* = 0.086	-	11 (24.4)	408 (13.0)	*p* = 0.024	-	
Parental tertiary education (*n*)	14 (34.1)	44 (72.1)	*p* < 0.001	-	16 (35.6)	1028 (32.8)	*p* = 0.693	-	
Physical activity(minutes/day)	162 (50)	172 (58)	*p* = 0.400	-	119 (59)	129 (73)	*p* = 0.334	-	
Mental health (mother-reported): Total Behavioral/emotional difficulties	7.34 (4.90)	7.36 (5.68)	*p* = 0.987	0.83	8.20 (5.95)	7.12 (5.40)	*p* = 0.187	0.71	0.129
Emotional symptoms	1.82 (1.85)	1.36 (1.67)	*p* = 0.206	0.70	2.36 (2.49)	1.84 (2.02)	*p* = 0.088	0.72	0.259 *
Conduct problems	1.25 (1.28)	1.64 (1.88)	*p* = 0.259	0.59	1.26 (1.64)	1.12 (1.38)	*p* = 0.488	0.62	−0.037
Hyperactivity/inattention	2.95 (2.23)	2.81 (2.47)	*p* = 0.776	0.80	2.69 (2.20)	2.60 (2.25)	*p* = 0.793	0.76	0.047
Peer problems	1.33 (1.42)	1.55 (1.75)	*p* = 0.499	0.57	1.89 (1.75)	1.56 (1.73)	*p* = 0.219	0.63	0.058
Well-being (child-reported): Overall well-being	126.08 (13.39)	125.75 (12.24)	*p* = 0.900	0.88	33.64 (7.64)	33.42 (6.48)	*p* = 0.815	-	0.031
Psychological well-being	20.43 (2.48)	20.34 (2.19)	*p* = 0.853	0.81	5.73 (1.53)	5.59 (1.40)	*p* = 0.483	-	0.079
Self-perception	17.37 (2.98)	16.95 (2.71)	*p* = 0.471	0.69	5.16 (1.54)	4.80 (1.55)	*p* = 0.132	-	0.203
Peer relations	19.68 (3.05)	18.90 (3.36)	*p* = 0.240	0.85	5.67 (1.55)	5.97 (1.28)	*p* = 0.118	-	−0.017
School related well-being	19.40 (2.70)	19.03 (3.24)	*p* = 0.558	0.82	11.02 (3.23)	11.00 (2.46)	*p* = 0.956	-	0.048

^1^ Pooled mean difference estimate from the random effects meta-analysis (See Supplementary Table S1); * *p* = 0.033.

**Table 2:** Meta-analysis of associations of physical activity as well as physical activity × preterm status interactions with behavioral/emotional difficulties and well-being

	Data Points	β	*p*	95% CI Lower Bound	95% CI Upper Bound	Cochran *Q* Test	*I* ^2^	Test for Heterogeneity (*p*)
Main Effects of Physical Activity								
Total behavioral/emotional difficulties	2	−0.017	0.819	−0.164	0.130	1.90	47.3	0.16
Emotional symptoms	2	−0.048	0.005	−0.081	−0.014	0.76	0.5	0.47
Conduct problems	2	0.007	0.938	−0.173	0.187	2.20	54.5	0.13
Hyperactivity/inattention	2	0.066	0.010	0.016	0.116	1.06	5.4	0.30
Peer problems	2	−0.086	<0.001	−0.120	−0.053	0.63	0.0	0.42
Overall well-being	2	0.057	0.001	0.022	0.091	0.17	0.0	0.67
Psychological	2	0.053	0.002	0.019	0.087	0.24	0.0	0.62
Self-perception	2	0.061	<0.001	0.028	0.094	0.32	0.0	0.57
Peer relations	2	0.014	0.440	−0.021	0.049	0.34	0.0	0.55
School related well-being	2	0.056	0.001	0.022	0.090	0.13	0.1	0.77
Interaction between Preterm status and Physical Activity								
Total behavioral/emotional difficulties	2	−0.244	0.084	−0.521	0.033	1.07	6.5	0.30
Emotional symptoms	2	−0.157	0.353	−0.426	0.112	0.22	0.0	0.64
Conduct problems	2	−0.127	0.366	−0.404	0.149	0.20	0.0	0.65
Hyperactivity/inattention	2	−0.281	0.046	−0.557	0.005	0.94	0.0	0.33
Peer problems	2	−0.182	0.183	−0.451	0.086	0.16	0.0	0.69
Overall well-being	2	0.070	0.842	−0.621	0.762	5.89	83.0	0.02
Psychological	2	−0.098	0.730	−0.651	0.456	4.05	75.3	0.04
Self-perception	2	−0.058	0.837	−0.604	0.489	3.77	73.5	0.05
Peer relations	2	0.052	0.713	−0.225	0.328	0.15	0.1	0.79
School related well-being	2	0.030	0.896	−0.416	0.475	2.61	61.8	0.10

Note: Dependent variables and physical activity were z-standardized before analyses; unstandardized regression coefficients are displayed.

**Table 3:** Hierarchical regressions of child- and mother-reported outcomes (z-standardized).

Heading	Basel Study of Preterm Children n = 40 Very Preterms (≤32nd Gest. Week); n = 59 Term Borns (≥37th Gest. Week)						Millennium Cohort Study n = 45 Very Preterms (≤32nd Gest. Week); n = 3137 Term Borns (≥37th Gest. Week)					
	Physical Activity 1			Preterm × PA			Physical Activity 1			Preterm × PA		
	β	*SE*	*p*	β	*SE*	*p*	β	*SE*	*p*	β	*SE*	*p*
Mental health (mother-rated SDQ): Overall behavioral and emotional difficulties	−0.167	0.111	0.136	−0.394	0.212	0.066	−0.012	0.016	0.459	−0.109	0.175	0.530
Emotional symptoms	−0.123	0.109	0.261	−0.225	0.211	0.289	−0.045	0.017	0.009	−0.096	0.178	0.590
Conduct problems	−0.166	0.119	0.168	−0.194	0.231	0.402	0.012	0.016	0.44	−0.067	0.169	0.690
Hyperactivity-inattention	−0.052	0.117	0.656	−0.427	0.224	0.061	0.069	0.017	<0.001	−0.150	0.175	0.391
Peer problems	−0.171	0.108	0.119	−0.240	0.210	0.255	−0.084	0.017	<0.001	−0.130	0.178	0.465
Well-being (child-rated): Overall well-being (scales combined)	0.105	0.117	0.378	0.444	0.226	0.053	0.055	0.017	0.002	−0.262	0.182	0.151
Psychological	0.105	0.110	0.341	0.201	0.215	0.352	0.051	0.017	0.003	−0.364	0.180	0.044
Self-perception	0.125	0.116	0.282	0.238	0.226	0.296	0.058	0.017	0.001	−0.320	0.177	0.072
Peer relations	−0.049	0.109	0.654	0.091	0.213	0.668	0.016	0.018	0.384	0.016	0.187	0.931
School related well-being	0.024	0.111	0.828	0.270	0.215	0.212	0.057	0.018	0.001	−0.185	0.182	0.309

Note: Dependent variables and physical activity were z-standardized before analyses; unstandardized regression coefficients are displayed. All models control preterm birth status, sex, age, sensory or motor impairments, ethnic minority status, and parental education. ^1^ Coefficients for physical activity from hierarchical regression models before the interaction term is entered to the model.

## Data Availability

Data of the BSPC can be accessed via: Department Klinische Forschung (Universität Basel) (DKF) https://dkf.unibas.ch/c/o (accessed on 8 February 2021) Universitätsspital Basel, Schanzenstrasse 55, 1. Stock, 4031 Basel, Switzerland. departement_klinische_forschung@usb.ch. Data of the Millennium Cohort Study are available through the UK Data Service, Center for Longitudinal Studies: https://cls.ucl.ac.uk (accessed on 8 February 2021) › cls-studies › millennium-cohort-study

## Supplementary Materials

The following are available online at https://www.mdpi.com/1660-4601/18/4/1735/s1, Table S1: Meta-analysis of differences in behavioral/ emotional difficulties and well-being between very preterm and full-terms. Table S2: Meta-analysis of associations of physical activity as well as physical activity x preterm status interactions with behavioral/ emotional difficulties and well-being without twins/triplets.
