# Supplementary material for: Physical Activity, Mental Health, and Well-Being in Very Pre-Term and Term Born Adolescents: An Individual Participant Data Meta-Analysis of Two Accelerometry Studies
Source: Int J Environ Res Public Health. 2021 Feb 10;18(4):1735. doi: 10.3390/ijerph18041735 (PMC7916780; doi:10.3390/ijerph18041735)
Supplement: Supplementary file 1 [file ijerph-18-01735-s001.pdf]

Table S1. Meta-analysis of differences in behavioral/ emotional difficulties and well-being between very preterm and full - terms.

	**Data Points**	**Std diff in means**	**95% CI Lower Bound**	**95% CI Upper Bound**	**Cochran *Q* Test**	**I^2**	**Test for Heterogeneity (*P*)**
Total Behavioral/ Emotional Difficulties	2	0.129	-0.109	0.366	0.642	0.000	0.288
Emotional symptoms	2	0.259	0.021	0.497	0.001	0.000	0.978
Conduct problems	2	-0.037	-0.361	0.286	1.737	42.420	0.188
Hyperactivity/Inattention	2	0.047	-0.191	0.284	0.006	0.000	0.941
Peer Problems	2	0.058	-0.257	0.372	1.646	39.250	0.199
Overall Well-Being	2	0.031	-0.206	0.268	0.001	0.000	0.975
Psychological	2	0.079	-0.159	0.316	0.058	0.000	0.810
Self-perception	2	0.203	-0.034	0.441	0.108	0.000	0.743
Peer relations	2	-0.017	-0.480	0.446	3.474	71.210	0.062
School related well-being	2	0.048	-0.190	0.285	0.201	0.000	0.654

Table S2. Meta-analysis of associations of physical activity as well as physical activity x preterm status interactions with behavioral/ emotional difficulties and well-being without twins/triplets.

	**Data Points**	β	***p***	**95% CI Lower Bound**	**95% CI Upper Bound**	**Cochran *Q* Test**	**I²**	**Test for Heterogeneity (*P*)**
Main Effects of Physical Activity								
Total Behavioral/ Emotional Difficulties	2	-0.011	0.501	-0.045	0.022	0.01	0.000	0.943
Emotional symptoms	2	-0.043	0.013	-0.077	-0.009	0.01	.000	0.908
Conduct problems	2	0.010	0.542	-0.022	0.042	0.08	0.000	0.772
Hyperactivity/Inattention	2	0.071	<0.001	0.038	0.105	0.05	0.000	.818
Peer Problems	2	-0.083	<0.001	-0.117	-0.049	0.06	0.000	0.808
Overall Well-Being	2	0.054	0.002	0.019	0.088	0.27	0.000	0.602
Psychological	2	.049	0.004	0.015	0.083	0.42	0.000	0.517
Self-perception	2	0.060	0.001	0.026	0.093	0.000	0.000	0.978
Peer relations	2	0.013	0.474	-0.022	0.048	0.50	0.000	0.480
School related well-being	2	0.056	0.001	0.022	0.091	0.18	0.000	0.670
Interaction between Preterm status and Physical Activity								
Total Behavioral/ Emotional Difficulties	2	-0.129	0.470	-0.480	0.221	0.30	0.000	0.582
Emotional symptoms	2	-0.068	0.705	-0.419	0.283	0.000	0.000	0.948
Conduct problems	2	-0.169	0.436	-0.594	0.256	1.28	22.0	0.258
Hyperactivity/Inattention	2	-0.174	0.344	-0.533	0.186	0.85	0.000	0.358
Peer Problems	2	-0.086	0.636	-0.444	0.271	0.02	0.000	0.895
Overall Well-Being	2	0.111	0.678	-0.413	0.635	1.92	48.0	0.165
Psychological	2	-0.118	0.567	-0.522	0.286	1.30	23.1	0.254
Self-perception	2	-0.074	0.706	-0.458	0.310	1.12	0.4	0.291
Peer relations	2	0.057	0.758	-0.307	0.421	0.15	0.000	0.695
School related well-being	2	0.067	0.721	-0.300	0.434	0.43	0.000	0.512
